# Editorial: Early intervention and prevention of severe mental illness: A child and adolescent psychiatry perspective

**DOI:** 10.3389/fpsyt.2022.963602

**Published:** 2022-07-07

**Authors:** Helen Minnis, Ruchika Gajwani, Dennis Ougrin

**Affiliations:** ^1^Institute of Health and Wellbeing, University of Glasgow, Glasgow, United Kingdom; ^2^Youth Resilience Unit, Queen Mary University of London, London, United Kingdom

**Keywords:** clinical staging model, developmental framework, child and adolescent psychiatry, transitions, public health, functioning, discrimination

Mental health services for adolescents and young adults are under extreme pressure and the increase in referrals to Child and Adolescent Mental health services since the start of COVID has worsened this (1)^1^. Yet only a small minority of young people with the poorest functioning and most complex psychiatric presentations are in contact with mental health services by age eighteen (2). Barriers to service delivery include missed diagnosis and poor early identification leading to widening social, health and occupational disadvantage (3).

The benefits of early detection and prevention of youth mental illness are well-known (4), but translation of research into scalable intervention has not succeeded. This may partly be due to the differences in diagnostic categorization and therapeutic models of service provision amongst child and adult services. For example, child services are less diagnostically driven, making the identification of specific at-risk groups (i.e., ADHD, at-risk of psychosis, mood disorders, and personality disorders) challenging (3). Lack of data about the transition between child and adult services limits knowledge about discontinuities in service provision for children, adolescents and young adults with most complex needs.

We propose an alternative model that borrows from other clinical specialties, and which is likely to make better use of scarce clinical resources: the **Developmental Clinical Staging Model**.

Clinical staging has been most effectively used in cancer services. International recommendations state that early-stage disease (i.e., with only local spread) should lead to the implementation of clear treatment plans in specialist centers with the aim of effecting cure and/or halting progression of disease (6, 7). Aggressive identification and treatment of Stages 1 or 2 breast cancers makes clinical and economic sense (8) since by Stage 4 (once distant metastases are established) it might not be possible to prolong healthy lifespan. Survival continues to improve even for cancers previously regarded as lethal (9). Careful disease staging and application of global treatment quality standards have undoubtedly contributed to this (9). Psychiatric services for young people, globally, take the opposite approach. Where mental health services exist at all, they focus only on young people with severe illness or risk of suicide (10). For those regarded as warranting specialist treatment, there has often been a long period of untreated illness: the equivalent of stages 1 and 2 were ignored and the opportunity for prolonging healthy lifespan may have been missed.

Here we propose a **Developmental Clinical Staging Model** (Table 1) to bridge the gap between child and adult services. We recommend three main areas of activity required to implement change:

1. Develop interventions that are scalable at a population level.
2. Focus on improving functional outcomes.
3. Actively engage the non-helping seeking children and young people.

**Table 1:** A Developmental Clinical Staging Model—preliminary structure for further discussion.

	**Psychiatric symptoms persisting for >3 months**	**Functional impairment**	**Rapid or significant deterioration in function**	**Risk to self or others**	**Greater likelihood of discrimination**
Stage 1	Yes	No	No	No	If any feature is “yes,” consider whether clinical stage has been underestimated
Stage 2	Yes or	Yes or	Yes	No	
Stage 3	Yes and	Yes and	Yes and	No	
Stage 4	Yes	Yes	Yes	Yes	

1. Scalable interventions: Previous attempts have been made to develop a clinical staging framework within adolescent mental health (11), however these have been limited to diagnoses within the adult taxonomy (12), such as psychosis and mania (13) and are expensive to implement. We are currently trialing a brief intervention that relies on careful clinical staging across disorders in both the child and adult taxonomies and on strengthening networks between existing clinical, educational and social services (https://clinicaltrials.gov/ *NCT05023447*).

2. Improve functional outcomes: Our research has shown that key early signs of severe psychiatric illness are *functional impairment and rate of change in functioning* (3). A common argument against early intervention in psychiatry is that, unlike cancer, psychiatric illness can be difficult to distinguish from less-than-optimal normal functioning. All of us, at certain points in our lives and in certain situations, will have these common experiences: sadness when grieving, loss of hope when we have just failed to get a job, physiological arousal when about to go for another interview. In fact, these experiences are easily distinguished from psychiatric symptoms by their transient nature and brief interference with daily functioning. Even experiences usually regarded as characteristic of severe mental illness, such as hearing voices, are more common in the population and less impairing than is often realized (14).

Change in functioning over time is a crucial early sign of mental illness in young people that is often ignored. One young person might slip into no longer attending school after years of low average functioning. Another young person might go from being top of the class to having low average school functioning. Both have the same rate of change in functioning. We have shown that investigating severe functional impairment or rapidly deteriorating functioning can be an important way of identifying severe mental illness at an early stage (3).

3. Engage non-help-seeking children and young people: The inverse care law, which states that “The availability of good medical care tends to vary inversely with the need for it” (15) has been clearly demonstrated in both psychiatry (15) and oncology (16). Individuals might recognize a health need but may have varying perceptions about “whether they as individuals …were entitled to care” (17). Stereotyping by clinicians, especially of individuals from ethnic minorities or with lower socioeconomic status, can compound this (18). Active engagement of young people across multiple sectors of society can result in more equitable service delivery (19).

Using these three key areas of focus as a guide, we have produced a **Developmental Clinical Staging Model**.

Clearly the details of this model and its implementation will require much discussion. Our key point is that the current approach to treating young peoples' psychiatric disorder is akin to setting up services specifically targeting only Stage 4 cancer, i.e., we focus almost entirely on those with entrenched problems, missing many opportunities for robust prevention and early intervention. At this time of global service scarcity, switching our priorities towards assessing and treating psychiatric disorder in young people at Stages 1 and 2 is likely to make excellent clinical and economic sense.

## Author contributions

All authors listed have made a substantial, direct, and intellectual contribution to the work and approved it for publication.

## Conflict of interest

The authors declare that the research was conducted in the absence of any commercial or financial relationships that could be construed as a potential conflict of interest.

## Publisher's note

All claims expressed in this article are solely those of the authors and do not necessarily represent those of their affiliated organizations, or those of the publisher, the editors and the reviewers. Any product that may be evaluated in this article, or claim that may be made by its manufacturer, is not guaranteed or endorsed by the publisher.

## Footnotes

^1^War and displacement are undoubtedly compounding this: at least 229 children have been killed and more than 424 injured since the beginning of the war in Ukraine; 12 million people have been displaced, with more than 6 million Ukrainian refugees, mainly women and children, fleeing to neighbouring countries (5).
